# Structural characterization reveals substrate recognition by the taurine transporter TauT

**DOI:** 10.1038/s41421-025-00785-1

**Published:** 2025-03-20

**Authors:** Hao Xu, Qinru Bai, Han Wang, Jun Zhao, Aiping Guo, Renjie Li, Qihao Chen, Yiqing Wei, Na Li, Zhuo Huang, Yan Zhao

**Affiliations:** 1 Division of Life Sciences and Medicine, University of Science and Technology of China, Hefei, Anhui China; 2 Key Laboratory of Biomacromolecules (CAS), National Laboratory of Biomacromolecules, CAS Center for Excellence in Biomacromolecules, Institute of Biophysics, Chinese Academy of Sciences, Beijing, China; 3 College of Life Sciences, University of Chinese Academy of Sciences, Beijing, China; 4 State Key Laboratory of Natural and Biomimetic Drugs, Department of Molecular and Cellular Pharmacology, School of Pharmaceutical Sciences, Peking University Health Science Center, Beijing, China; 5 Peking University Institute of Advanced Agricultural Sciences, Shandong Laboratory of Advanced Agricultural Sciences at Weifang, Weifang, Shandong China; 6 Heart Center and Beijing Key Laboratory of Hypertension, Beijing Chaoyang Hospital, Capital Medical University, Beijing, China

**Keywords:** Cryoelectron microscopy, Molecular biology

Dear Editor,

Taurine, an organic acid abundant in animal tissues, is crucial for various physiological processes in humans, particularly in the brain, heart, retina, and skeletal muscles^1^. Although the body can synthesize taurine, production often falls short, necessitating exogenous supplementation. The taurine transporter (TauT, SLC6A6) plays a vital role in taurine absorption and distribution, facilitating its transport across cell membranes^2^.

Dysfunction of TauT can lead to reduced taurine levels, contributing to conditions such as retinopathy and cardiomyopathy^3^. Additionally, TauT is overexpressed in certain cancers, and is correlated with poor prognosis and aggressive tumor behavior^4^. Inhibiting TauT may offer therapeutic potential; however, current inhibitors have not proven effective in treating cancer. Therefore, gaining more structural insights into TauT could facilitate the development of targeted drugs, ultimately advancing this potential therapy for cancer treatment.

We employed cryo-EM single-particle reconstruction to determine the structure of TauT in complex with taurine (TauT^TAU^), β-alanine (TauT^BAL^), and in a substrate-free state (TauT^APO^), achieving the resolutions of 2.8 Å, 3.2 Å, and 2.9 Å, respectively (Fig. 1a; Supplementary Figs. S1–S3 and Table S1). These structures enabled us to unambiguously resolve the side chains of residues, elucidate sterols and lipids, and characterize substrate–transporter interactions in TauT. Detailed information related to cryo-EM sample preparation, data collection and processing, as well as model building, is provided in Supplementary Materials and Methods.

**Fig. 1:**
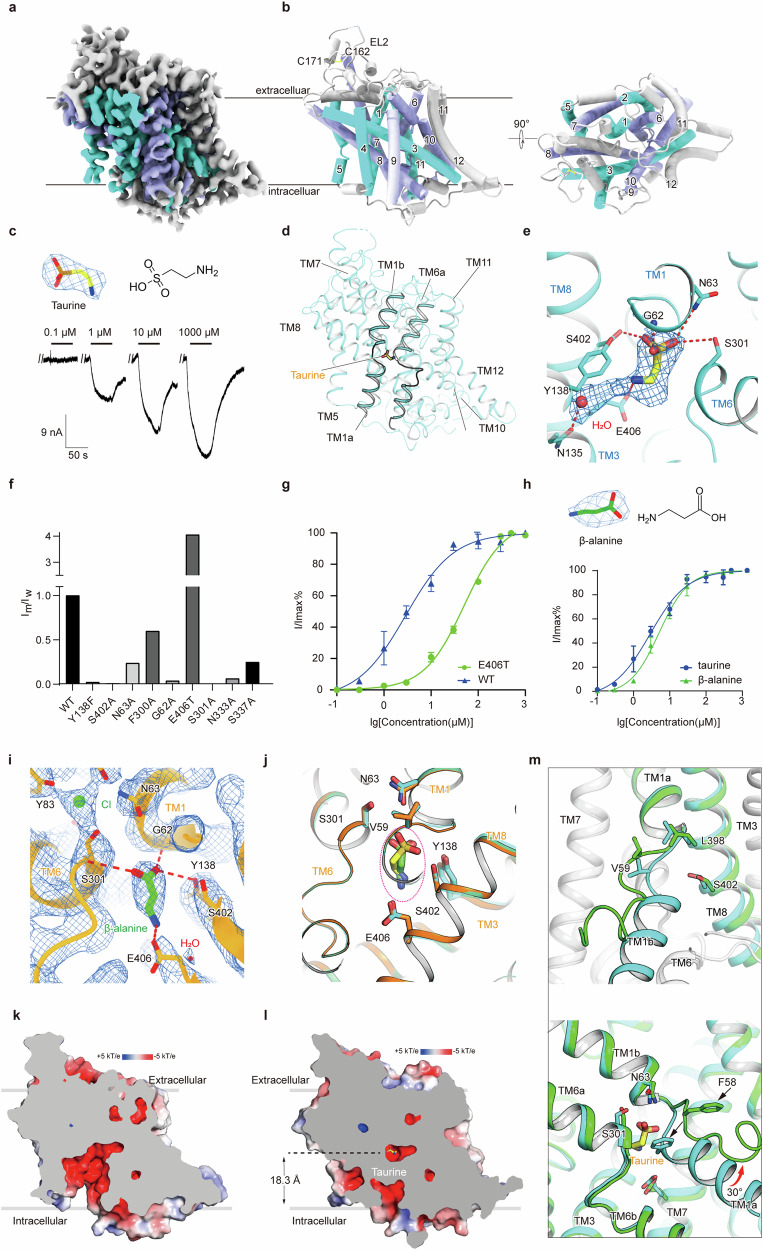
Structural basis of TauT binding with the substrate taurine, the inhibitory substrate β-alanine, and the apo state. **a**, **b** The cryo-EM density map of TauT^TAU^ along with the atomic model, displays TM1–TM5 in cyan, TM6–TM10 in purple, and other regions in gray. **c** Taurine (yellow sticks) is depicted with its corresponding EM density (blue mesh), and the chemical structure is shown on the right. Leak currents from the taurine concentration–response curves were measured in the same *X. laevis* oocyte expressing the human TauT protein (bottom). **d** The structure of TauT^TAU^ is show with TM1 and TM6 highlighted. **e** The binding pose of taurine in combination with TauT. The taurine (shown as yellow sticks) and the coordinated water molecule (depicted as a red sphere) are displayed with their corresponding EM density (blue mesh). **f** The functional activity of TauT^WT^ and mutations was measured in *X. laevis* oocytes. The taurine-induced inward current ratio (I_m_/I_w_) was normalized based on band density quantification using ImageJ. **g** Concentration response curves of TauT^WT^ and TauT^E406T^. **h** β-alanine (green sticks) is depicted with its corresponding EM density (blue mesh), and its chemical structure is shown on the right. Concentration–response curves for TauT^WT^ induced by taurine and β-alanine are shown below. **i** The binding pose of β-alanine in complex with TauT. **j** Superimposition of the substrate-binding pockets between TauT^TAU^ (cyan) and TauT^BAL^ (orange). Taurine is represented by yellow sticks, while β-alanine is shown in green. **k**, **l** The surface electrostatic potential of the TauT^APO^ (**k**) and TauT^TAU^ (**l**) are depicted in a cut-open view. **m** Magnified superimposed view of the TM1 structure in TauT^TAU^ and TauT^APO^.

TauT belongs to solute carrier family 6 (SLC6), which comprises 12 transmembrane helices connected by extracellular and intracellular loops (ELs and ILs). Both the N-terminus and C-terminus are located on the cytoplasmic side, adopting a pseudo-symmetric topology characteristic of SLC proteins, with an inverted pseudo-twofold symmetry between TM1–TM5 and TM6–TM10^5^ (Fig. 1a, b). TM1 and TM6 both exhibit partial unwinding in the middle, which, together with TM3 and TM8, contributes to the formation of the substrate-binding pocket. Additionally, within EL2, a disulfide bond exists between the conserved Cys171 and Cys162 residues (Supplementary Fig. S4). Two mutations in TauT, A78E and G399V, have been shown to significantly reduce TauT transport activity, leading to retinal lesions (Supplementary Figs. S4, S6)^3^. We mapped these pathogenic mutations onto our TauT structure, revealing that A78E is located on TM2, at the interface between the extracellular fluid and the cell membrane. The substitution of glutamic acid for alanine introduces an additional charge, which may impact the folding of TauT. G399V is located on TM8, which engages in the formation of the central binding pocket. This mutation replaces the non-polar glycine residue with the hydrophobic side chain of valine, potentially causing steric clashes with the side chains of nearby amino acids. Additionally, the elimination of glycine increases the rigidity of the helix, which may hinder the conformational changes required for TauT to transport taurine (Supplementary Fig. S6).

Under physiological pH conditions, taurine behaves as an amphipathic molecule. We designate the sulfonic acid group of taurine as the “head”, with the remainder, including the positively charged amine, referred to as the “tail”. Previous studies have shown that the substrate taurine is transported by TauT in a stoichiometric ratio of 2 Na^+^:1 Cl^–^:1 taurine. This electrically coupled transport of Na^+^/Cl^–^ and taurine allows us to record taurine-induced, concentration-dependent inward currents using a two-electrode voltage clamp in *Xenopus* *laevis* oocytes expressing wild-type TauT^5,6^ (Fig. 1c). The EC_50_ of taurine was determined by exposing the oocytes to various concentrations of taurine, ranging from 0.1 μM to 10 mM. The results revealed an EC_50_ value of 2.9 μM, confirming that the recombinantly expressed TauT retained its transport function. The taurine molecule is located within a negatively charged central pocket formed by TM1a, TM1b, TM6a, TM6b, TM3, and TM8 (Fig. 1d, e). This pocket is situated 18.3 Å from the cytoplasmic side. The transmembrane segments TM1a, TM6b, and TM8 form a barrier around the central pocket, sealing it off from the cytoplasm. Moreover, TM1b, TM6a, TM8, and TM10 obstruct access from the extracellular environment, resulting in an occluded conformation (Fig. 1l).

The residues in the taurine-bound pocket extensively participate in the polar interactions with the head sulfonic acid group of taurine. Specifically, the main chain of residue G62 and the side chains of residues N63, Y138, S301, and S402 form hydrogen bonds with the sulfonic acid group of taurine (Fig. 1d, e). To investigate the effects of these positions on TauT transport activity, we constructed corresponding mutants by substituting the residues with alanine. The alanine substitution at G62 may create steric hindrance with taurine, and enhance the rigidity of the helix. The other mutants eliminate the polar side chains that are capable of forming hydrogen bonds with taurine. Electrophysiological experiments demonstrated that these mutations substantially reduced the transport activity of TauT compared with that of the wild type (Fig. 1f; Supplementary Fig. S7).

The positively charged amine group of taurine can form a salt bridge with the negatively charged side chain of residue E406 located on TM8. We replaced E406 with threonine (TauT^E406T^) to disrupt this crucial electrostatic interaction. The TauT^E406T^ mutant showed an increased EC_50_ of 50.3 μM, compared with 2.9 μM for the wild type, suggesting its important role in substrate recognition. These factors underscore the critical function of residue E406 in stabilizing taurine binding. Additionally, we observed that the inward current in the E406T mutant is significantly increased at saturating taurine concentrations, suggesting that this residue may also be important for the transport cycle of TauT^7^ (Fig. 1f, g).

TauT is implicated in various physiological and pathological processes, prompting interest in the molecules that can reduce TauT’s transport of taurine. The most effective competitive substrate identified is the taurine analog β-alanine, which differs from taurine by having a carboxyl group instead of a sulfonic group^8^. Owing to their structural similarity, β-alanine can also be transported by TauT. In *X.* *laevis* oocytes, β-alanine induces an inward current with an EC_50_ of 5.6 µM, which is slightly less than that of taurine, likely due to the larger volume of the sulfonic group allowing for better charge distribution and interaction (Fig. 1h–j).

The SLC6 family primarily transports small molecules, including amino acids, neurotransmitters, energy metabolites and osmolytes, typically with molecular weights of less than 200 Da. GABA, glycine, and taurine share similar chemical structures, each featuring a negatively charged acidic head group and a positively charged amino tail. However, they are transported by different members of the SLC6 family, with varying substrate preferences^5^. Although some evidence suggests that members of other SLC families can transport taurine, their affinity for taurine is significantly lower than that of TauT. The structural comparison between TauT and GAT1 (PDB: 7Y7W)^9^ or GlyT1 (PDB: 8WFI)^10^ indicates that the residues interacting with the acidic head groups of taurine (carboxyl group) and glycine (sulfonyl group) are conserved. However, there are significant differences in the residues surrounding the substrate tail. In GlyT1, the residues Y62, W322, and T418 are replaced by G57, L306, and S402 in TauT. This substitution of amino acids with smaller side chains enlarges the central pocket of TauT, facilitating the binding of larger substrates (Supplementary Figs. S4, S5). The previous research found that GABA binds to TauT with a low affinity of 1018 μM^11^, which is two orders of magnitude lower than that of taurine. By comparing the structural characteristics of the central substrate-binding pockets of GAT1 and TauT, we discovered that a key aspect of this interaction is the characteristic salt bridge formed between E406 in TauT and the positively charged tail of the substrate. For GABA, a longer tail may interfere with E406, which is equivalent to a threonine with a smaller side chain in GAT1 (Supplementary Figs. S4, S5).

Additionally, the SLC6 family also encompasses the monoamine transporters (MATs), which include the dopamine transporter^12^, norepinephrine transporter^13^ and serotonin transporter. These transporters play crucial roles in regulating synaptic activity by recycling neurotransmitters in the synaptic cleft^5^. The substrates transported by MATs are characterized by an aromatic ring at the head and a positively charged amine at the tail. For MATs, electrostatic interactions are also crucial for substrate binding; however, unlike E406 in TM8 of TauT, the acidic residues within the central binding pockets of MATs are located in TM1 to coordinate the amino group of monoamine neurotransmitters. Moreover, monoamines typically possess an aromatic ring that fits into the cavity between TM3 and TM8, close to three glycine residues. However, in TauT, these residues are replaced by the polar residues N135, Q403, and E406. This structural difference prevents TauT from uptaking monoamine substrates (Supplementary Figs. S4, S5).

The substrate transport mechanism of secondary active transporters follows the “alternating access” model^14^, where the protein shifts between inward-facing, occluded, and outward-facing states. We characterized the substrate-free TauT^APO^ in an inward-open state, revealing a solvent-accessible central pocket (Fig. 1k), unlike the occluded state (Fig. 1l). By comparing TauT^TAU^ and TauT^APO^, we analyzed the transition from outward-facing to inward-facing states that facilitates substrate release. During this process, TM1a acts as an intracellular gate, unwinding and shifting 30 degrees and creating a cavity that opens to the cytoplasm, allowing substrate release (Fig. 1m).

In conclusion, our research elucidates the substrate binding mechanisms of TauT, as well as the conformational changes involved. These findings provide a foundation for further understanding the transport of taurine within the body. Additionally, by studying the binding pattern of the competitive substrate and the differences in the central substrate-binding pocket across various conformations, we lay the groundwork for the development of TauT-targeted inhibitors, which could provide potential therapeutic interventions for cancer.

## Supplementary information

Supplementary Information

## Data Availability

The cryo-EM density maps for TauT^TAU^, TauT^BAL^, and TauT^APO^ have been submitted to the Electron Microscopy Data Bank with accession codes EMD-61207, EMD-61208, and EMD-61209, respectively. The coordinates for TauT^TAU^, TauT^BAL^, and TauT^APO^ are available in the Protein Data Bank under accession codes 9J7M, 9J7N, and 9J7O, respectively.
