# Supplementary material for: Structural characterization reveals substrate recognition by the taurine transporter TauT
Source: Cell Discov. 2025 Mar 20;11:28. doi: 10.1038/s41421-025-00785-1 (PMC11923213; doi:10.1038/s41421-025-00785-1)
Supplement: Supplementary file 1 — Supplementary Information [file 41421_2025_785_MOESM1_ESM.pdf]

Supplementary information

Structural characterization reveals substrate recognition by the taurine transporter TauT

Hao Xu[1,2]*, Qinru Bai[2,3]*, Han Wang[4]*, Jun Zhao[5]*, Aiping Guo[4], Jieren Li[2,3], Qihao Chen[2, 3], Yiqing Wei[2,3], Na Li[6]**, Zhuo Huang[4]**, Yan Zhao[2,3]**

[1] Division of Life Sciences and Medicine, University of Science and Technology of China, Hefei, Anhui, China

[2] Key Laboratory of Biomacromolecules (CAS), National Laboratory of Biomacromolecules, CAS Center for Excellence in Biomacromolecules, Institute of Biophysics, Chinese Academy of Sciences, Beijing 100101, China

[3] College of Life Sciences, University of Chinese Academy of Sciences, Beijing 100049, China

[4] State Key Laboratory of Natural and Biomimetic Drugs, Department of Molecular and Cellular Pharmacology, School of Pharmaceutical Sciences, Peking University Health Science Center, Beijing, 100191, China

[5] Peking University Institute of Advanced Agricultural Sciences, Shandong Laboratory of Advanced Agricultural Sciences at Weifang, Weifang, China

[6] Heart Center and Beijing Key Laboratory of Hypertension, Beijing Chaoyang Hospital, Capital Medical University, Beijing, 100020, China.

* These authors contribute equally to this project.

** Correspondence email: zhaoy@ibp.ac.cn (Y.Z.), huangz@hsc.pku.edu.cn (Z.H.), linapumc@126.com (N.L.)

This document contains Supplementary figure S1-S7 and Table S1.

This pdf file includes:

Materials and Methods

Supplementary Fig. S1 to S7

Supplementary Table S1

Materials and Methods

Protein expression and purification

The human Taurine transporter protein (UniPro ID P31641-1) gene was cloned from the HEK 293 cDNA library. Subsequently, the wild-type or mutant fragment was subcloned into a pEG BacMam vector (Invitrogen) with a PreScission Protease (PPase) recognition site (LEVLFQ/GP), followed by a C-terminal superfolder GFP (sfGFP) tag and a Twin-Strep affinity tag. All mutations were generated by the polymerase chain reaction (PCR)-based site-directed mutagenesis method. The Bac-to-Bac baculovirus expression system was employed to produce efficient recombinant baculovirus, which was used to transfect HEK-293F cells for expressing above fusion protein. The cells were suspended in a shaking incubator supplemented with 1% (v/v) fetal bovine serum, and incubated at 37°C with 5% CO_2. A final concentration of 10 mM sodium butyrate was added to cultures to induce protein expression at 30°C. After 48 hours, cells were collected by centrifugation at 3000 rpm for 3 minutes at 4°CThe supernatant was removed, and the cell pellets were flash-frozen, then thawed on ice in buffer A (20 mM Tris-HCl, pH 8.0, 150 mM NaCl, 5 mM β-mercaptoethanol) supplemented with 2 μg/mL aprotinin (MedChemExpress), 1.4 μg/mL leupeptin (MedChemExpress), and 0.5 μg/mL pepstatin A (MedChemExpress). Dounce homogenizers were used to disrupt cells, and membrane fractions were collected by centrifugation at 35,000 rpm for 30 minutes. The membrane pellet was gently resuspended and incubated with 1% (w/v) n-dodecyl β-D-maltoside (DDM) and 0.15% (w/v) cholesteryl hemisuccinate (CHS) (Anatrace, USA) on a rotating platform for 2 hours at 4°C to solubilize the protein from the membrane. After centrifugation at 36,000 rpm for 30 minutes, the supernatant was loaded onto Streptactin Beads 4FF (Smart-Lifesciences) for purification at 4°C. The column was washed with 3 volumes of buffer B (buffer A supplemented with 0.025% DDM, 0.15% CHS, 5 mM $MgCl_2$, and 2 mM ATP). Buffer C (Buffer A supplemented with 5 mM d-desthiobiotin (1169249, Leyan), 0.025% DDM, 0.15% CHS) was used to competitively elute the protein. The protein fraction was concentrated to less than 1 mL using a 50-kDa Millipore Tube (Merck Millipore) and purified by size-exclusion chromatography on a Superdex 6 Increase 10/300 GL column (GE Healthcare, USA), pre-equilibrated with buffer D (buffer A supplemented with 0.025% DDM,

0.15% CHS, and 10 μM taurine or β-alanine). Protein fractions were further separated and collected using an AKTA system. The collected fractions were then concentrated to less than 1 mL with a 50-kDa Millipore Tube (Merck Millipore) for subsequent nanodisc reconstitution.

Lipid preparation and nanodiscs reconstitution

The total brain polar lipid (BTL) (BPL, Avanti) was dissolved in chloroform to create a homogeneous mixture and vacuumed overnight to remove residual organic solvent until a dry lipid film formed. This film was then resuspended in basic buffer (20 mM Tris, 150 mM NaCl, pH 8.0) and the multilamellar vesicles (LMVs) were disrupted via multiple freezing and thawing in liquid nitrogen and ultrasound, yielding smaller unilamellar vesicles. By squeezing the liposomes into a filter membrane with a pore size of 0.22 μm, a lipid suspension of homogeneous and monodisperse was obtained. This lipid was used for subsequent reconstitution. The concentrated protein was incubated with BTL and scaffold protein MSP1D1 at a molar ratio of 1:5:100 for 1 hour at 4°C with gentle shaking. Detergent removal was performed by adding 400 mg/mL Bio-Beads (SM2, Bio-Rad) for 2h. A fresh portion of Bio-Beads was then incubated with the supernatant for 2 hours, followed by a third replacement and overnight incubation. Holes were punched in the tube wall to enable the separation of recombinant protein components from the Bio-Beads through centrifugation.

The supernatant was then loaded onto streptactin beads 4FF (Smart-Lifesciences) and washed with buffer A to remove empty nanodiscs, followed by buffer E (buffer A supplemented with 5 mM d-desthiobiotin to elute the protein. Before loading onto the Superose 6 Increase 10/300 GL column, the sfGFP-StrepII tag of the TauT protein was digested with PPase and removed by passing the sample over His-Pur Ni-NTA resin (Thermo Fisher Scientific) in the presence of 10 mM imidazole at 4°C. The flowthrough was collected and further purified by size-exclusion chromatography on a Superdex 6 Increase 10/300 GL column, pre-equilibrated with buffer A. The peak fractions were concentrated to approximately 12 mg/mL. The 10 mM β-alanine or 10 mM taurine was incubated with the concentrated protein for 30 min before applied to the freshly glow-discharged grids.

Cryo-EM sample preparation and data acquisition

Quantifoil 1.2/1.3 Cu 300 mesh grids were glow discharged for 60 sec using a Solarus plasma cleaner (Gatan, USA) in the H2-O2 condition. Applied with 2.5 μL protein droplet to the freshly glow-discharged grid and blot for 4.5 s in 100% humidity at 4°C. Then the grid was flash-frozen in liquid ethane cooled by liquid nitrogen with Vitrobot Mark IV (Thermo Fisher Scientific). Cryo-EM data were collected on a 300-kV Titan Krios G4 (Thermo Fisher Scientific, UAS) equipped with a Gatan K3 Summit detector (Gatan). The slit width was set to 10 eV. Movie stacks were acquired using EPU software in super-resolution mode at a calibrated magnification of 105,000x, resulting in a raw pixel size of 0.425 Å, with defocus values ranging from -1.0 to -2.0 μm. The movie stacks were dose-fractionated into 32 frames, resulting in a total accumulated dose of 60 e^-/Å², with a dose rate set to 15 s.

Cryo-EM data processing

A total of 1569/1314/3357 cryo-EM movie were collected for $TauT^{TAU}$, $TauT^{BAL}$ and $TauT^{APO}$ respectively. For $TauT^{TAU}$ database, the movies were imported into CryoSPARC and performed dose-weighting by Patch Motion Correction, followed by patch-CTF estimation. Following this, 2,718,237 particles of interest were selected from the micrographs using automated particle picking (blob picker). After 3-4 rounds of ab-initio reconstruction and two rounds of 2D classification, poor-quality particles were removed, and an initial model at a resolution of 3.06 Å was generated using 159,692 particles, which served as a 'seed' for further processing. Then three bias maps were imported along with the initial map to remove bad particles by heterogeneous refinement. To avoid discarding good protein particles, the raw particles were divided randomly into 3 groups, each containing 262,7000 particles. Each group of particles were combined with the 'seed' particles and the potential good particles were retrieved from the original dataset through several rounds of seed-facilitated 3D classification. New discrete data classes with distinct spiral features in 2D class average are enriched. And the final map was refined to 2.9 Å through local refinement using 146,104 particles. Detailed procedures are exemplified by the $TauT^{AU}$ dataset processing (Supplementary Fig. S2a). A similar strategy was applied to process the $TauT^{BAL}$ and $TauT^{APO}$

data, and the final maps were refined to 3.2 Å for $TauT^{BAL}$ and 2.8 Å for $TauT^{APO}$ through local refinement, utilizing 66,563 and 271,080 particles, respectively.

Model building and refinement

To build the atomic models of TauT, the predicted AlphaFold2(AF2) model of TauT (AF-P31641-F1) was manually fitted into the cryo-EM density map of TauT using UCSF Chimera 1.16[45]. The initial model was inspected and adjusted in COOT, both main-chain and side-chain residues are manually fitted in the corresponding cryo-EM density map. Geometry restraint file for Taurine was generated using elBOW module in PHENIX[47], and the final atomic models were adjusted in COOT. The structure was then refined in real-space with secondary structure and Ramachandran restraints using 'phenix.real space refine' tool. All three TauT models underwent secondary structure and geometry restraint corrections in real space with PHENIX[48]. All figures were prepared with PyMOL 2.4.0 (Schrödinger, LLC, https://pymol.org/), and UCSF ChimeraX 1.7.1. The surface electrostatic potential of TauT was calculated using APBS plugin. Fourier shell correlation (FSC) curves for the 'Model vs Map' were generated using 'Phenix.real_space_refine' from Phenix. The summary of the cryo-EM data collection and model refinement statistics was shown on Table S1.

The electrophysiological studies in *Xenopus* Oocytes

Oocytes were collected from anesthetized Xenopus laevis female clawed frogs and subsequently washed twice with a Ca^{2+}-free OR2 solution (82.5 mM NaCl, 2.5 mM KCl, 1 mM $MgCl_2$, 5 mM HEPES, pH 7.4). The oocytes were then transferred to approximately 25 mL tubes and treated with 2 mg/mL collagenase (Sigma type II, Sigma-Aldrich Inc., St Louis, MO, USA) in OR2 solution for 45 minutes at 20−25 °C with gentle rotation. They were then washed twice with OR2 solution followed by three washes with ND96 solution (96 mM NaCl, 2 mM KCl, 1 mM $MgCl_2$, 1.8 mM $CaCl_2$, 5 mM HEPES, pH 7.4). Stage V and VI oocytes were then selected for microinjections.

For two-electrode voltage clamp recordings, capped cRNAs were synthesized *in vitro* using the T3 mM ESSAGEmMACHINE Kit (Ambion, Austin, TX, USA) after the linearization

of plasmids within pBluescript KSM vectors. A total of 32 nL of cRNA solution, containing approximately 64 ng of either wild-type TauT or its mutant variants, was injected into each oocyte. The oocytes were then incubated for 3-5 days at 16 °C in ND96 solution supplemented with 0.1 g/L gentamycin. Oocytes were impaled with two microelectrodes (0.5−1.5 MΩ) filled with 3 M KCl in a 40-μL recording chamber. The membrane potential was maintained at −100 mV using standard voltage clamp techniques. Currents were recorded at room temperature (22 ± 1 °C) in ND96 solution, utilizing a GeneClamp 500B amplifier (Axon Instruments, Union City, CA). The superfusion flow rate was 10 mL/min, enabling a complete exchange of the bath solution in about 10 seconds. The recorded currents represent the peak values observed during a 20-second substrate superfusion. The GABA transport activity of each mutant was normalized to that of $TauT^{WT}$. The number of Xenopus oocytes used to measure GABA transport activity was: WT (n = 12), Y138F (n = 10), N63A (n = 8), F300A (n = 7), G62A (n = 6), E406T (n = 12), and S301A (n = 6). The ratio I_m / I_w represents the mean currents of the Taut mutations compared to the wild type from the same batch of *Xenopus oocytes*. Each experiment was performed with at least two or three independent batches of oocytes, consistently yielding qualitatively similar results. To account for potential variations in protein expression, the data were further normalized using cell surface biotinylation experiments conducted in 293F cells. Western blot (WB) analysis and band density measurements using ImageJ were then performed, with the wild-type (WT) serving as the reference for expression levels. For concentration-response curves, 12 oocytes were used for WT and 9 for the E406T mutation. Data are presented as means ± SEM (error bars), with 'n' representing the number of oocytes examined. Each experiment was performed with at least two or three different batches of oocytes, consistently producing qualitatively similar results.

Cell-surface biotinylation and internalization assay

The wild-type SLC6A6 gene and its relevant mutations were subcloned into the pEG BacMan vector, which includes GFP and FLAG purification tags at the C-terminus. Utilizing the Bac-to-Bac baculovirus system, viral pellets were generated and added to HEK-293F

cells with a virus-to-cell volume ratio of 1:100. The transfected cells were cultured at 37°C in an environment with 5% CO_2 for 12 hours, followed by the addition of 1 mM sodium butyrate to boost protein expression. After 48 hours, cell fluorescence was monitored using a fluorescence microscope, and a cell count was performed. The 3×10^6 cells were collected by centrifugation at 1000 rpm for 3 minutes, and the pellets were washed twice with precooled HEPES buffer (150 mM NaCl, 20 mM HEPES, pH 7.5). The cells were then resuspended in 800 μL of HEPES buffer and incubated with sulfo-NHS-SS-biotin at a final concentration of 1 mM at 4°C for 30 minutes to perform biotinylation. The cells were then resuspended in 800 μL of HEPES buffer and incubated with sulfo-NHS-SS-biotin at a final concentration of 1 mM at 4°C for 30 minutes to perform biotinylation. For membrane protein extraction, the cell pellets were resuspended in 800 μL of lysis buffer containing HEPES buffer supplemented with 2 μg/mL aprotinin, 1.4 μg/mL leupeptin, 0.5 μg/mL pepstatin A, and 1% DDM (wt/vol). The suspension was then centrifuged at 15,000 rpm for 30 minutes at 4°C. Biotinylated membrane proteins were purified using 70 μL of Streptactin Beads 6FF (Smart-Lifesciences). To remove non-specifically bound proteins, 2 mL of wash buffer (20 mM HEPES, 150 mM NaCl, 2 μg/mL aprotinin, 1.4 μg/mL leupeptin, 0.5 μg/mL pepstatin A, and 0.025% DDM) was added. The Streptactin Beads 6FF beads were resuspended in 80 μL of wash buffer and centrifuged at 13,000 rpm for 30 minutes. The suspension was then analyzed by SDS-PAGE and immunoblotting. Protein bands density was quantified using ImageJ, and the expression levels of wild-type TauT and its mutants were determined.

Supplementary Figures

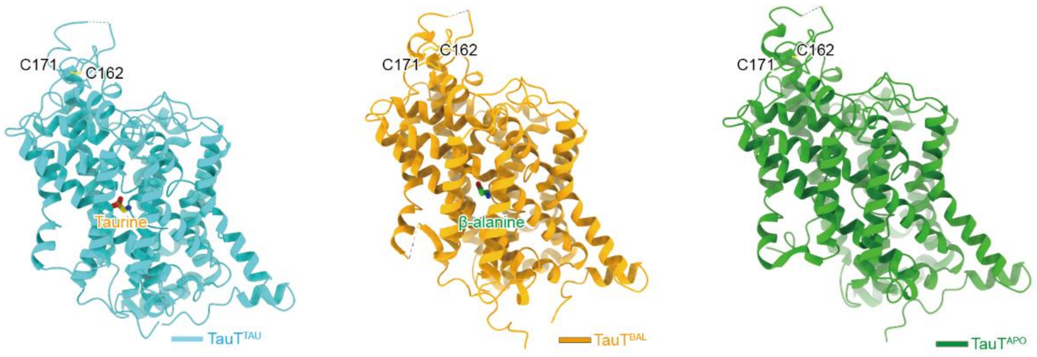

Supplementary information, Figure S1. The structures of human TauT.

Overall structures of TauT binding with taurine (Tau^{TAU}), β-alanine (Tau^{BAL}) and substrate-free state ($TuaT^{APO}$) are represented by cyan, orange, green respectively. Taurine and β-alanine are depicted as yellow and green sticks, respectively. The cysteine residues in extracellular loop 2 that are involved in the formation of disulfide bonds are highlighted in yellow and labeled.

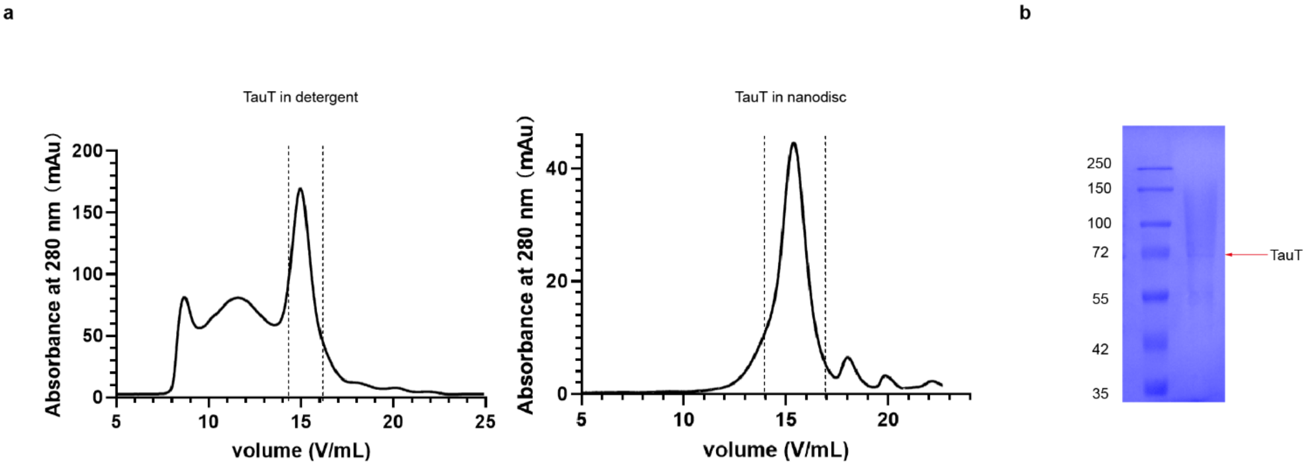

Supplementary information, Figure S2. Purification of human TauT.

a. The characteristic size-exclusion chromatography (Superose 6 increase) profile of TauT proteins in detergent (left) or reconstituted in nanodiscs (right). Peak fractions between black dashed lines are collected and concentrated for nanodisc-reconstitution (left panel) or cryo-EM sample preparation (right panel), respectively. **b.** The cryo-EM sample of TauT is visualized by coomassie blue-stained SDS-PAGE gel. The target components of the reconstituted TauT-nanodisc complex are annotated. The experiments are conducted independently more than three times.

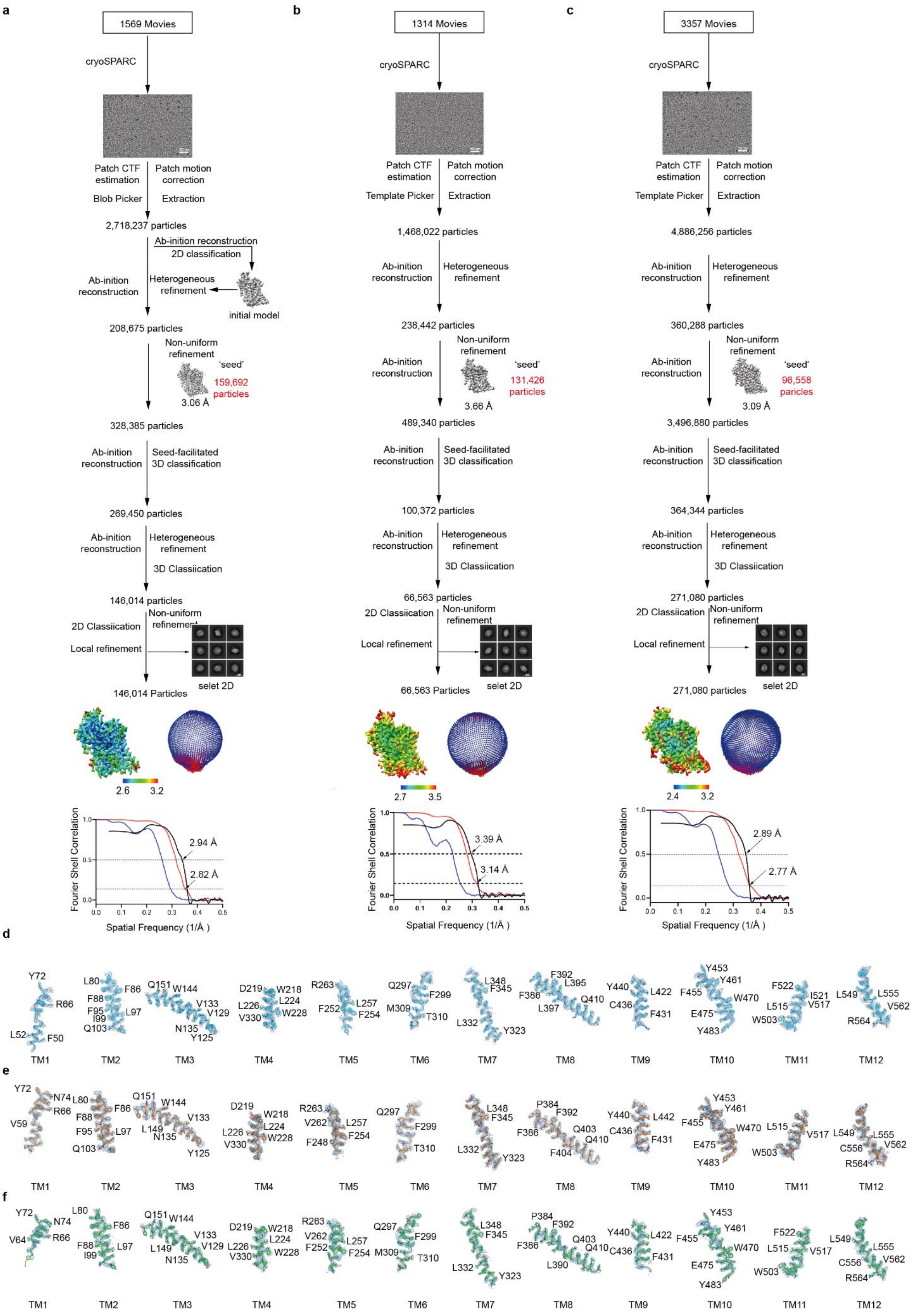
a
1569 Movies
cryoSPARC
Patch CTF estimation
Patch motion correction
Blob Picker
Extraction
2,718,237 particles
Ab-inition reconstruction
2D classification
Ab-inition reconstruction
Heterogeneous refinement
initial model
208,675 particles
Non-uniform refinement
'seed'
159,692 particles
3.06 Å
328,385 particles
Ab-inition reconstruction
Seed-facilitated 3D classification
269,450 particles
Ab-inition reconstruction
Heterogeneous refinement
3D Classiication
146,014 particles
Non-uniform refinement
2D Classiication
Local refinement
146,014 Particles
selet 2D
2.6
3.2
Fourier Shell Correlation
2.94 Å
2.82 Å
Spatial Frequency (1/Å)
b
1314 Movies
cryoSPARC
Patch CTF estimation
Patch motion correction
Template Picker
Extraction
1,468,022 particles
Ab-inition reconstruction
Heterogeneous refinement
238,442 particles
Non-uniform refinement
Ab-inition reconstruction
'seed'
131,426 particles
3.66 Å
489,340 particles
Ab-inition reconstruction
Seed-facilitated 3D classification
100,372 particles
Ab-inition reconstruction
Heterogeneous refinement
3D Classiication
66,563 particles
2D Classiication
Non-uniform refinement
Local refinement
66,563 Particles
selet 2D
2.7
3.5
Fourier Shell Correlation
3.39 Å
3.14 Å
Spatial Frequency (1/Å)
c
3357 Movies
cryoSPARC
Patch CTF estimation
Patch motion correction
Template Picker
Extraction
4,886,256 particles
Ab-inition reconstruction
Heterogeneous refinement
360,288 particles
Non-uniform refinement
Ab-inition reconstruction
'seed'
96,558 paricles
3.09 Å
3,496,880 particles
Ab-inition reconstruction
Seed-facilitated 3D classification
364,344 particles
Ab-inition reconstruction
Heterogeneous refinement
3D Classiication
271,080 particles
2D Classiication
Non-uniform refinement
Local refinement
271,080 particles
selet 2D
2.4
3.2
Fourier Shell Correlation
2.89 Å
2.77 Å
Spatial Frequency (1/Å)
d
TM1 TM2 TM3 TM4 TM5 TM6 TM7 TM8 TM9 TM10 TM11 TM12
e
TM1 TM2 TM3 TM4 TM5 TM6 TM7 TM8 TM9 TM10 TM11 TM12
f
TM1 TM2 TM3 TM4 TM5 TM6 TM7 TM8 TM9 TM10 TM11 TM12

Supplementary information, Figure S3. Cryo-EM data processing of TauT.

a-c. Flowchart for cryo-EM data processing of TauT combined to taurine (**a.** $TauT^{TAU}$), β-alanine (**b.** $TauT^{BAL}$), and substrate-free (**c.** $TauT^{APO}$) conditions. Several rounds of heterogeneous and ab-initio refinement are employed to sort and remove poor-quality particles, followed by non-uniform and local refinement to improve image quality. The scale bar for representative 2D class averages is 9 nm. The angular distribution of particles used in the final reconstruction is shown and Gold-standard Fourier shell correlation (FSC) plot (masked and unmasked) is generated by comparing two independently refined half-maps before (blue) and after (red) post-processing, using an FSC cut-off of 0.143. According to the FSC criterion mentioned above, the final cryo-EM maps are reported at 2.9 Å for $TauT^{TAU}$, 3.2 Å for $TauT^{BAL}$, and 2.8 Å for $TauT^{APO}$, respectively. The FSC curve comparing the cryo-EM map to the model is depicted in black, with a cut-off of 0.5. Detailed data processing steps are provided in the Methods section. **d-f.** The cryo-EM densities superimposed with the atomic features of TM1-12 in $TauT^{TAU}$ (cyan), $TauT^{BAL}$ (orange), $TauT^{APO}$ (green).

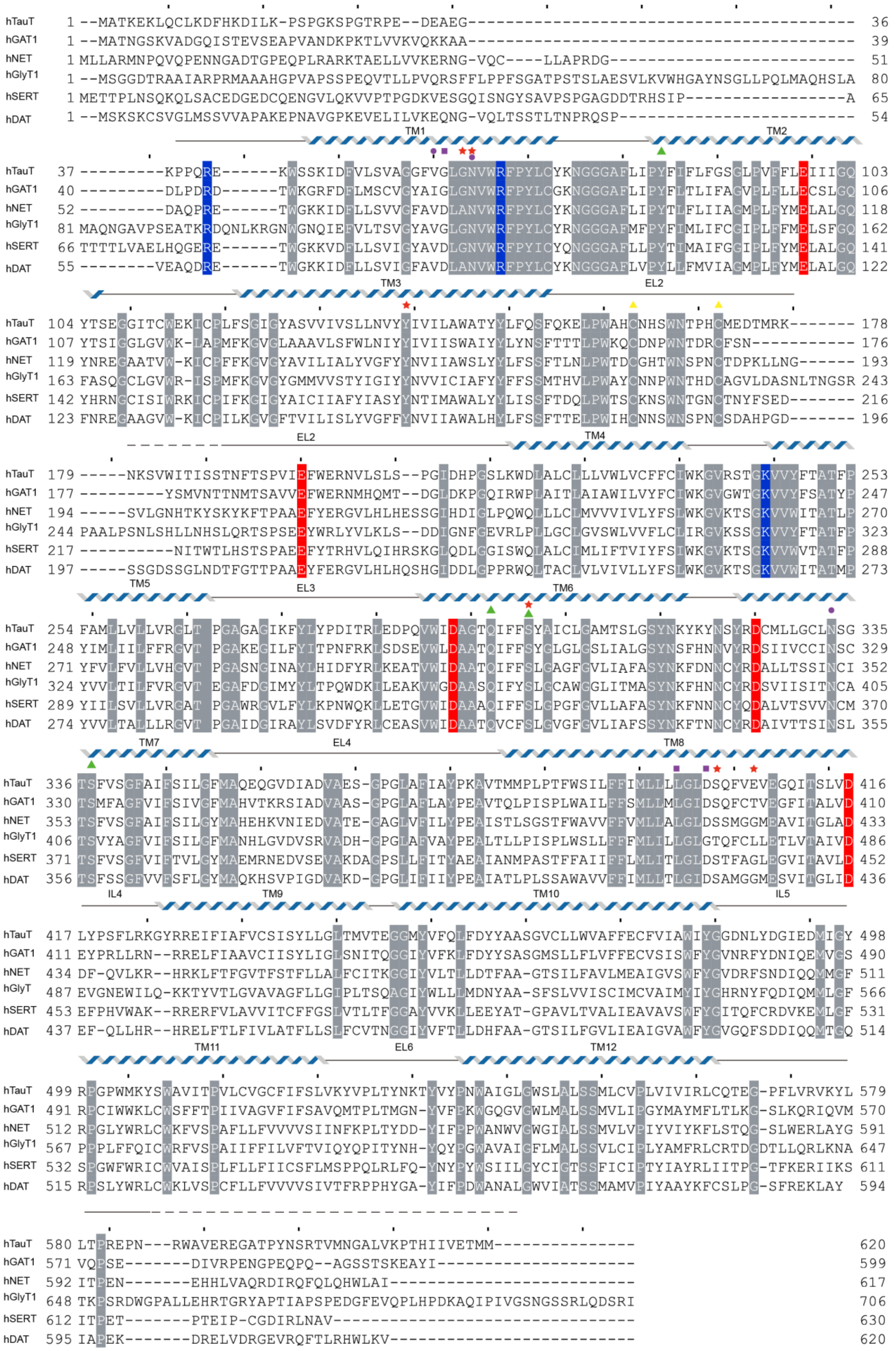

hTauT 1 --MATKEKLQCLKDFHKDILK-PSPGKSPGTRPE--DEAEG-- 36
hGAT1 1 --MATNGSKVADGQISTEVSEAPVANDKPKTLVVKVQKKAA-- 39
hNET 1 MLLARMNPQVQPENNGADTGPEQPLRARKTAELLVVKERNG-VQC----LLAPRDG---------------------------- 51
hGlyT1 1 --MSGGDTRAAIARPRMAAAHGPVAPSSPEQVTLLPVQRSFFLPPFSGATPSTSLAESVLKVWHGAYNSGLLPQLMAQHSLA 80
hSERT 1 METTPLNSQKQLSACEDGEDCQENGVLQKVVPTPGDKVESGQISNGYSAVPSPGAGDDTRHSIP-----------------A 65
hDAT 1 --MSKSKCSVGLMSSVVAPAKEPNAVGPKEVELILVKEQNG-VQLTSSTLTNPRQSP-------------------------- 54
TM1
TM2
hTauT 37 ---------KPPQRE------KWSSKIDFVLSVAGGFVGLGNVWRFPYLCYKNGGGAFLIPYFIFLFGSGLPVFFLEIIIGQ 103
hGAT1 40 ---------DLPDRD------TWKGRFDFLMSCVGYAIGLGNVWRFPYLCGKNGGGAFLIPYFLTLIFAGVPLFLLECSLGQ 106
hNET 52 ---------DAQPRE------TWGKKIDFLLSVVGFAVDLANVWRFPYLCYKNGGGAFLIPYTLFLIIAGMPLFYMELALGQ 118
hGlyT1 81 MAQNGAVPSEATKRDQNLKRGNWGNQIEFVLTSVGYAVGLGNVWRFPYLCYRNGGGAFMFPYFIMLIFCGIPLFFMELSFGQ 162
hSERT 66 TTTTLVAELHQGERE------TWGKKVDFLLSVIGYAVDLGNVWRFPYICYQNGGGAFLLPYTIMAIFGGIPLFYMELALGQ 141
hDAT 55 --------VEAQDRE------TWGKKIDFLLSVIGFAVDLANVWRFPYLCYKNGGGAFLVPYLLFMVIAGMPLFYMELALGQ 122
TM3
EL2
hTauT 104 YTSEGGITCWEKICPLFSGIGYASVVIVSLLNVYYIVILAWATYYLFQSFQKELPWAHCNHSWNTPHCMEDTMRK------- 178
hGAT1 107 YTSIGGLGVWK-LAPMFKGVGLAAAVLSFWLNIYYIVIISWAIYYLYNSFTTTLPWKQCDNPWNTDRCFSN----------- 176
hNET 119 YNREGAATVW-KICPFFKGVGYAVILIALYVGFYYNVIIAWSLYYLFSSFTLNLPWTDCGHTWNSPNCTDPKLLNG------ 193
hGlyT1 163 FASQGCLGVWR-ISPMFKGVGYGMMVVSTYIGIYYNVVICIAFYYFFSSMTHVLPWAYCNNPWNTHDCAGVLDASNLTNGSR 243
hSERT 142 YHRNGCISIWRKICPIFKGIGYAICIIAFYIASYYNTIMAWALYYLISSFTDQLPWTSCKNSWNTGNCTNYFSED------- 216
hDAT 123 FNREGAAGVW-KICPILKGVGFTVILISLYVGFFYNVIIAWALHYLFSSFTTELPWIHCNNSWNSPNCSDAHPGD------- 196
EL2
TM4
hTauT 179 -----NKSVWITISSTNFTSPVIEFWERNVLSLS--PGIDHPGSLKWDLALCLLLVWLVCFFCIWKGVRSTGKVVYFTATFP 253
hGAT1 177 ---------YSMVNTTNMTSAVVEFWERNMHQMT--DGLDKPGQIRWPLAITLAIAWILVYFCIWKGVGWTGKVVYFSATYP 247
hNET 194 -----SVLGNHTKYSKYKFTPAAEFYERGVLHLHESSGIHDIGLPQWQLLLCLMVVVIVLYFSLWKGVKTSGKVVWITATLP 270
hGlyT1 244 PAALPSNLSHLLNHSLQRTSPSEEYWRLYVLKLS--DDIGNFGEVRLPLLGCLGVSWLVVFLCLIRGVKSSGKVVYFTATFP 323
hSERT 217 ----------NITWTLHSTSPAEEFYTRHVLQIHRSKGLQDLGGISWQLALCIMLIFTVIYFSIWKGVKTSGKVVWVTATFP 288
hDAT 197 -----SSGDSSGLNDTFGTTPAAEYFERGVLHLHQSHGIDDLGPPRWQLTACLVLVIVLLYFSLWKGVKTSGKVVWITATMP 273
TM5
EL3
TM6
hTauT 254 FAMLLVLLVRGLTPGAGAGIKFYLYPDITRLEDPQVWIDAGTQIFFSYAICLGAMTSLGSYNKYKYNSYRDCMLLGCLNSG 335
hGAT1 248 YIMLIILFFRGVTPGAKEGILFYITPNFRKLSDSEVWLDAATQIFFSYGLGLGSLIALGSYNSFHNNVYRDSIIVCCINSC 329
hNET 271 YFVLFVLLVHGVTPGASNGINAYLHIDFYRLKEATVWIDAATQIFFSLGAGFGVLIAFASYNKFDNNCYRDALLTSSINCI 352
hGlyT1 324 YVVLTILFVRGVTEGAFDGIMYYLTPQWDKILEAKVWGDAASQIFYSLGCAWGGLITMASYNKFHNNCYRDSVIISITNCA 405
hSERT 289 YIILSVLLVRGATPGAWRGVLFYLKPNWQKLLETGVWIDAAAQIFFSLGPGFGVLLAFASYNKFNNNCYQDALVTSVVNCM 370
hDAT 274 YVVLTALLLRGVTPGAIDGIRAYLSVDFYRLCEASVWIDAATQVCFSLGVGFGVLIAFSSYNKFTNNCYRDAIVTTSINSL 355
TM7
EL4
TM8
hTauT 336 TSFVSGFAIFSILGFMAQEQGVDIADVAES-GPGLAFIAYPKAVTMMPLPTFWSILFFIMLLLLGLDSQFVEVEGQITSLVD 416
hGAT1 330 TSMFAGFVIFSIVGFMAHVTKRSIADVAAS-GPGLAFLAYPEAVTQLPISPLWAILFFSMLLMLGIDSQFCTVEGFITALVD 410
hNET 353 TSFVSGFAIFSILGYMAHEHKVNIEDVATE-GAGLVFILYPEAISTLSGSTFWAVVFFVMLLALGLDSSMGGMEAVITGLAD 433
hGlyT1 406 TSVYAGFVIFSILGFMANHLGVDVSRVADH-GPGLAFVAYPEALTLLPISPLWSLLFFFMLILLGLGTQFCLLETLVTAIVD 486
hSERT 371 TSFVSGFVIFTVLGYMAEMRNEDVSEVAKDAGPSLLFITYAEAIANMPASTFFAIIFFLMLITLGLDSTFAGLEGVITAVLD 452
hDAT 356 TSFSSGFVVFSFLGYMAQKHSVPIGDVAKD-GPGLIFIIYPEAIATLPLSSAWAVVFFIMLLTLGIDSAMGGMESVITGLID 436
IL4
TM9
TM10
IL5
hTauT 417 LYPSFLRKGYRREIFIAFVCSISYLLGLTMVTEGGMYVFQLFDYYAASGVCLLWVAFFECFVIAWIYGGDNLYDGIEDMIGY 498
hGAT1 411 EYPRLLRN--RRELFIAAVCIISYLIGLSNITQGGIYVFKLFDYYSASGMSLLFLVFFECVSISWFYGVNRFYDNIQEMVGS 490
hNET 434 DF-QVLKR--HRKLFTFGVTFSTFLLALFCITKGGIYVLTLLDTFAA-GTSILFAVLMEAIGVSWFYGVDRFSNDIQQMMGF 511
hGlyT 487 EVGNEWILQ-KKTYVTLGVAVAGFLLGIPLTSQAGIYWLLLMDNYAA-SFSLVVISCIMCVAIMYIYGHRNYFQDIQMMLGF 566
hSERT 453 EFPHVWAK--RRERFVLAVVITCFFGSLVTLTFGGAYVVKLLEEYAT-GPAVLTVALIEAVAVSWFYGITQFCRDVKEMLGF 531
hDAT 437 EF-QLLHR--HRELFTLFIVLATFLLSLFCVTNGGIYVFTLLDHFAA-GTSILFGVLIEAIGVAWFYGVGQFSDDIQQMTGQ 514
TM11
EL6
TM12
hTauT 499 RPGPWMKYSWAVITPVLCVGCFIFSLVKYVPLTYNKTYVYPNWAIGLGWSLALSSMLCVPLVIVIRLCQTEG-PFLVRVKYL 579
hGAT1 491 RPCIWWKLCWSFFTPIIVAGVFIFSAVQMTPLTMGN-YVFPKWGQGVGWLMALSSMVLIPGYMAYMFLTLKG-SLKQRIQVM 570
hNET 512 RPGLYWRLCWKFVSPAFLLFVVVVSIINFKPLTYDD-YIFPPWANWVGWGIALSSMVLVPIYVIYKFLSTQG-SLWERLAYG 591
hGlyT1 567 PPPLFFQICWRFVSPAIIFFILVFTVIQYQPITYNH-YQYPGWAVAIGFLMALSSVLCIPLYAMFRLCRTDGDTLLQRLKNA 647
hSERT 532 SPGWFWRICWVAISPLFLLFIICSFLMSPPQLRLFQ-YNYPYWSIILGYCIGTSSFICIPTYIAYRLIITPG-TFKERIIKS 611
hDAT 515 RPSLYWRLCWKLVSPCFLLFVVVVSIVTFRPPHYGA-YIFPDWANALGWVIATSSMAMVPIYAAYKFCSLPG-SFREKLAY 594
hTauT 580 LTPREPN---RWAVEREGATPYNSRTVMNGALVKPTHIIVETMM--------------- 620
hGAT1 571 VQPSE-------DIVRPENGPEQPQ--AGSSTSKEAYI--------------------- 599
hNET 592 ITPEN-------EHHLVAQRDIRQFQLQHWLAI-------------------------- 617
hGlyT1 648 TKPSRDWGPALLEHRTGRYAPTIAPSPEDGFEVQPLHPDKAQIPIVGSNGSSRLQDSRI 706
hSERT 612 ITPET-------PTEIP-CGDIRLNAV-------------------------------- 630
hDAT 595 IAPEK-------DRELVDRGEVRQFTLRHWLKV-------------------------- 620

Supplementary information, Figure S4. Multiple sequence alignment of SLC6 transporters.

a. Sequence alignment of human TauT (SLC6A6, UniProt: P31641-1), human GAT1 (SLC6A1, UniProt: P30531-1), human NET (SLC6A2, UniProt: P23975-1), human GlyT1 (SLC6A9, UniProt: P48067-3), human SERT (SLC6A4 UniProt: P31645-1), human DAT (SLC6A3, UniProt: Q01959) is performed using ClustalW and vualized with Jalview. Conserved acidic and basic residues are highlighted in red and blue, respectively, while other conserved residues are shaded in grey. The residues involved in the taurine binding pocket are marked with red pentagrams while the conserved cysteine residues in EL2 are indicated with yellow arrows. The binding sites for Na1, Na2, and Cl ions in the SLC6 family are represented by purple circles, purple squares, and green triangles, respectively.

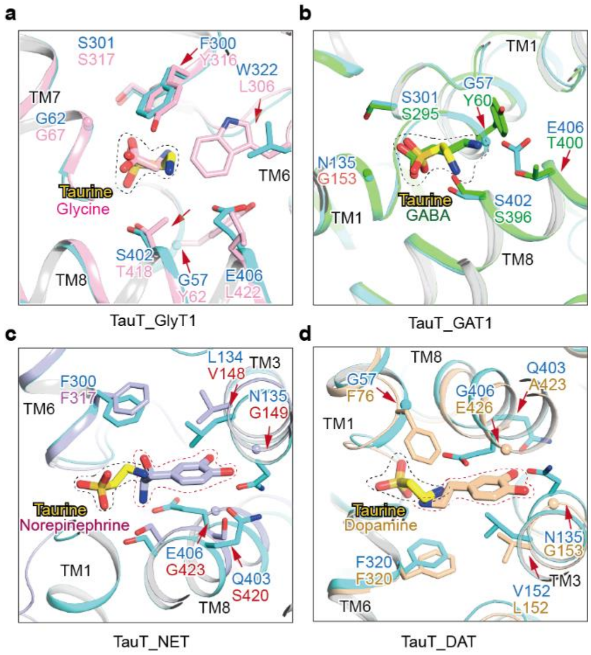

Supplementary information, Figure S5. Substrate-binding pocket structure alignment between TauT with SLC6 sub-families.

a-d: Structural alignment of the substrate binding sites of TauT (cyan) with glycine bound to human GlyT1 (pink) (PDB: 8WFI), GABA bound to human GAT1 (green) (PDB: 7Y7W), norepinephrine bound to human NET (lightblue) (PDB: 8WTV), and dopamine bound to human DAT (orange) (PDB: 8Y2D). Taurine, glycine, norepinephrine, GABA, and dopamine are represented as yellow, light pink, purple, and orange sticks, respectively. Residues involved in substrate binding are labeled, with arrowheads highlighting key residues that may determine substrate selectivity. The substrate binding pocket is outlined with black and red dashed lines

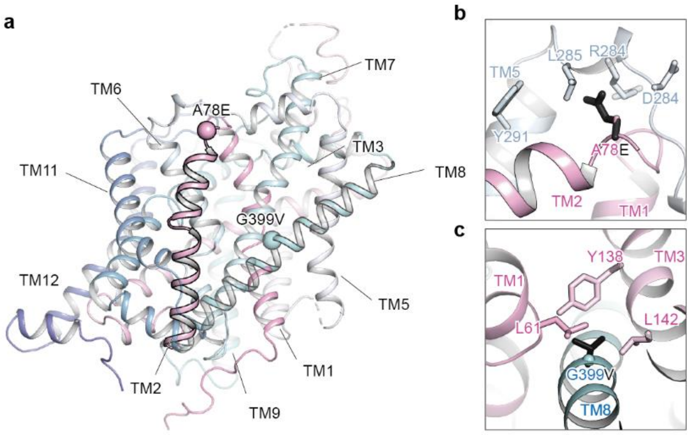

Supplementary information, Figure S6. Disease-related mutations are mapped on TauT.

a. The disease-related mutations are mapped on TauT, with pink and cyan spheres representing mutations associated with retinal degeneration and cardiomyopathy in children. The TauT structure is colored with a gradient from lightblue at the N-terminus to pink at the C-terminus. **b-c.** The location of the pathogenic point mutations and the surrounding residues are shown in an enlarged view. Mutated residues at the corresponding locations are depicted as black sticks.

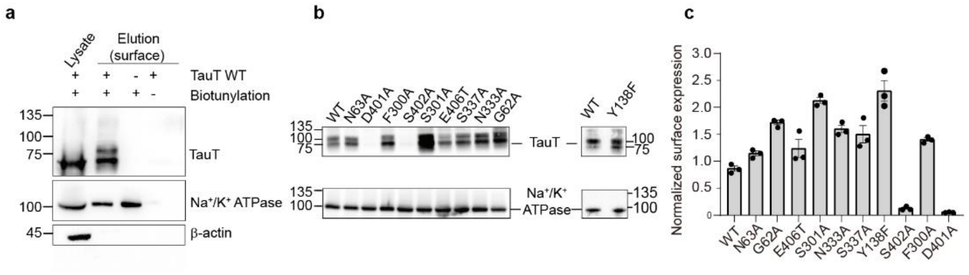

‘Supplementary information, Figure S7. Surface expression of TauT ^{WT} and mutants

a. Biotinylation system validation. β-actin was detected only in the cell lysate from biotinylated HEK-293F cells that expressing TauT protein. While Na^+/K^+ ATPase was detected in all biotinylated samples, indicating that biotinylation occurs primarily at the cell surface. TauT was detected only in the lysate from biotinylated HEK-293F cells expressing TauT, as well as in the biotinylated cell membrane samples from these cells. This confirming that TauT on the cell membrane can be effectively biotinylated, purified using streptavidin beads, and detected by anti-Flag antibody. **b**. Detection of cell surface-expressed TauT WT and mutants by Western blot. Biotinylated Na^+/K^+ ATPase on the cell surface was used as a loading control. **c**. Densitometric analysis of TauT WT and mutant surface expression was performed in HEK-293F cells, with normalization using the density values of Na^+/K^+ ATPase within each experimental batch, as well as the mean density values across independent batches. Data are mean ± S.E.M. of n = 3 independent experiments.

Supplementary Table S1 Cryo-EM data collection, refinement and validation statistics.

	$TauT^{APO}$ (EMD-38850) (PDB 8Y2C)	$TauT^{TAU}$ (EMD-38851) (PDB 8Y2D)	$TauT^{BAL}$ (EMD-38852) (PDB 8Y2E)
**Data collection and processing**			
Magnification	×105,000	×105,000	×105,000
Voltage (kV)	300	300	300
Electron exposure ($e^{-}/Å^2$)	60	60	60
Defocus range (μm)	−1.0 – −2.0	−1.0 – −2.0	−1.0 – −2.0
Pixel size (Å)	0.85	0.85	0.85
Symmetry imposed	C1	C1	C1
Initial particle images (no.)	4,677,646	1,163,338	1,063,973
Final particle images (no.)	271,080	146,014	146,014
Map resolution (Å)	2.9	3.0	3.4
FSC threshold	0.143	0.143	0.143
**Refinement**			
Model resolution (Å)	2.8	2.9	3.2
FSC threshold	0.5	0.5	0.5
Map sharpening *B* factor ($Å^2$)	-109.8	-116.1	-120.6
Model composition			
Non-hydrogen atoms	4,344	4,552	4,533
Protein residues	533	543	546
Ligands	6	12	12
B factors ($Å^2$)			
Protein	48.27	50.86	101.18
Ligand	42.41	67.16	107.43
R.m.s. deviations			
Bond lengths (Å)	0.008	0.005	0.006
Bond angles (°)	0.750	0.677	0.741
Validation			
MolProbity score	1.63	1.70	1.94
Clashscore	3.10	3.31	6.46
Poor rotamers (%)	0.00	0.00	0.00
Ramachandran plot			
Favored (%)	97.92	97.03	95.18
Allowed (%)	2.08	2.97	4.64
Disallowed (%)	0.00	0.00	0.00
